# Review Article

**Published:** 2008-11

**Authors:** Derick Van Vuuren, Amanda Lochner

**Affiliations:** Department of Biomedical Sciences, Division of Medical Physiology, Faculty of Health Sciences, University of Stellenbosch; Department of Biomedical Sciences, Division of Medical Physiology, Faculty of Health Sciences, University of Stellenbosch

## Abstract

**Summary:**

The increase in the incidence of ischaemic heart disease and acute myocardial infarction (AMI) in both high- and low-income countries necessitates the development of myocardial salvaging/protection interventions, to be applied alongside standard reperfusion therapies. Although the phenomenon of ischaemic preconditioning (IPC) is associated with the desired protective capacity, the necessity of its application before sustained ischaemia limits its clinical potential.

The recently described phenomenon of postconditioning (postC), or short cycles of reperfusion/ischaemia applied at the onset of reperfusion, falls within the clinically relevant time period of reperfusion, but can it elicit reliable and potent cardioprotection? The answer to this problem is intimately related to the question whether postC can be translated from a laboratory technique to a clinical therapy.

In this brief overview of postconditioning, the experimental set-ups and postC algorithms utilised, and their associated outcomes in all animal models studied (dog, rabbit, mouse, rat and pig) are discussed. The therapeutic potential of postC is also addressed by discussing reported preliminary studies on the efficacy and feasibility of postC (both ischaemic and pharmacological) in humans.

## Summary

Ischaemic heart disease is one of the leading causes of mortality, especially in the developed world. According to projections,^1^,^2^ ischaemic heart disease is set to remain a major contributor to global mortality rates in high-, middle- and low-income countries. Since the duration of ischaemia is one of the most important factors determining the extent of ischaemic damage,^3^ rapid reperfusion is critical in the treatment of an unexpected myocardial ischaemic incident, namely, an acute myocardial infarction (AMI). Despite the utilisation of effective reperfusion strategies such as thrombolytic treatment and percutaneous coronary intervention (PCI), there is still a need for the development of interventions to increase tissue viability during ischaemia and reperfusion.^4^ It is in this setting of reperfusion adjunct therapies that postconditioning (postC) is of potential importance.

## Postconditioning

In 1986, Murry and colleagues^5^ made the surprising discovery that multiple, brief episodes of ischaemia, applied before a sustained ischaemic insult, did not contribute to ischaemic injury, but rather induced an increased tolerance against ischaemic damage. This phenomenon, coined ischaemic preconditioning (IPC), has proven to be the most robust and potent intervention to confer protection against ischaemia/reperfusion.^6^ The fact that IPC has to be administered before the onset of ischaemia has unfortunately minimised the clinical applicability of this intervention.

However, it has recently been shown that a similar intervention applied at the very onset of reperfusion also substantially limits ischaemia/reperfusion injury.^7^ Termed postconditioning, this intervention is defined as the application of brief cycles of reperfusion/ischaemia at the onset of reperfusion, eliciting cardioprotection against ischaemia/reperfusion injury.^8^ Targeting of reperfusion events by the application of an intervention during reperfusion, which elicits a reduction in damage (such as postC), is viewed as proof of the existence of reperfusion injury *per se* (ie, a separate entity from ischaemic damage).^9^

Postconditioning is clinically more relevant than IPC, since it constitutes a potent natural protective mechanism that can be triggered during the clinically applicable time period of reperfusion. It is therefore not surprising that, since its description in 2003,^7^ much research has been done on the topic. Although postC has been demonstrated in all species studied (dog, rabbit, mouse, rat and pig), there are contradictions and uncertainties as to the precise postC algorithm that is the best to apply.

The aim of this review is to give a critical overview of the different postC protocols and algorithms (as well their associated outcomes) that have been reported in the different animal models studied, with the aim of identifying some of the factors that influence postC in the experimental setting. Following this evaluation of postC in the laboratory setting, the potential of postC in the clinical setting will also be discussed.

## From the bench: postconditioning in the laboratory

## The canine model

Postconditioning was first described in the *in vivo* dog heart, with an infarct-sparing effect comparable to IPC.^7^ In this model, a postC protocol of three cycles of 30 sec (3 × 30 sec) reperfusion and ischaemia was also associated with a decrease in neutrophil accumulation in the area at risk (AAR), preserved coronary endothelial function and a reduction in reactive oxygen species (ROS) generation and oxidative damage. The efficacy of this 3 × 30-sec protocol to reduce damage has also been shown by others.^10^,^11^ Despite these positive findings, Couvreur and coworkers^12^ could not show an anti-stunning effect for postC in the canine heart, even though they applied various protocols similar to the 3 × 30-sec protocol (4 × 15, 30 or 60-sec reperfusion/ischaemia, applied after 10 min regional ischaemia).

Fujita and colleagues^13^ followed a completely different protocol by applying a 90-min period of regional ischaemia (contrary to the 60 min used by others in the canine model), followed by a postC protocol of 4 × 60-sec reperfusion/ischaemia. Despite these differences, they could also illustrate a postC-mediated decrease in infarct size. The canine heart can therefore readily be protected against infarct development by the application of a postconditioning intervention, although a beneficial effect on functional recovery remains to be shown.

## The rabbit heart

The positive outcomes found in the initial canine study^7^ could also be replicated in the next species to be postconditioned: the rabbit. Yang *et al*.^14^ reported a 43% decrease in infarct size, comparable to the infarct-sparing effects of IPC, with a postC protocol of either four or six cycles of 30 sec of reperfusion/ischaemia in an *in vivo* model. Interestingly, they found that the postC intervention could still be protective, even if it was applied after 10 min of reperfusion. On the other hand, Downey and Cohen^15^ reported that postC had to be implemented within one minute of reperfusion in their rabbit model. Other researchers^12^,^16^ similarly found that a 4 × 30-sec protocol could decrease infarct size, although it did not exert an anti-stunning effect after 10 min coronary occlusion.^12^

Yang and co-workers^17^ went on to demonstrate postconditioning in the isolated rabbit heart, illustrating that at least a measure of the observed protection was due to intrinsic mechanisms of the heart, independent of blood-borne factors. Interestingly, they found that the 4 × 30-sec protocol used in the *in vivo* model was less beneficial than a more rapid protocol of 6 × 10 sec of reperfusion/ischaemia – contrary to Darling and colleagues^18^ who found a 4 × 30-sec protocol adequate to elicit an infarct-sparing effect in their isolated rabbit heart model.

Other studies have also shown a protective role for postC in the rabbit heart, even with the administration of different protocols (Table 1). The reported infarct-sparing effects of postC, despite differences in algorithm, suggest that postC protection is robust in the rabbit heart.

**Table 1:** Several Different Post-C Protocols Applied In Similar In Vivo Rabbit Heart Experiments, As Well As Three Different Protocols In The Mouse Heart, Demonstrating The Experimental Variability Of Post-C

	*Experimental set-up*					*Outcome*				
		*Ischaemia*					*Results*			
*Authors*	*Model*		*Type*		*Duration*		*Reperfusion duration*		*PostC protocol*		*Parameter*		*Control (%)*		*PostC (%)*		*Difference (%)*		*Conclusion*	
***Postconditioning in the rabbit***										
Iliodromitis *et al.*19	*In vivo*	RI	30 min	180 min	4 × 30 sec	IFS	48.2 ± 4.3	45.1 ± 8.9	NS	Non-protective
					6 × 10 sec			20.4 ± 2.9	↓ 57.7	Protective
Chiari *et al.*20	In vivo	RI	30 min	180 min	3 × 10 sec	IFS	41 ± 2	34 ± 3	NS	Non-protective
					3 × 20 sec			20 ± 3	↓ 51	Protective
Argaud *et al.*21	*In vivo*	RI	30 min	240 min	4 × 60 sec	IFS	61 ± 6	29 ± 4	↓ 52	Protective
				72 h			48 ± 6	20 ± 5	↓ 58	Protective
***Unique mouse heart postconditioning protocols***										
Yang *et al.*22	*In vivo*	RI	40 min	60 min	3 × 5 sec	IFS	51 ± 2	37 ± 3	↓ 27	Protective
Tsutsumi *et al.*23	*In vivo*	RI	30 min	120 min	3 × 20 sec	IFS	43.4 ± 3.3	24.1 ± 3.2	↓ 44	Protective
						RPP	Pre-ischaemic: 29.9 ± 2.6	27.5 ± 2.9	NS	Maintained heart function
Gomez *et al.*24	*In vivo*	RI	60 min	24 h	3 × 60 sec	IFS	56 ± 5	39 ± 3	↓ 30	Protective

RI: regional ischaemia; IFS: infarct size; RPP: rate pressure product (beats.min^-1^.mmHg.10^-3^); NS: non-significant.

## The mouse heart

Primarily two postC protocols have been described for the mouse heart, namely 3 × 10 sec and 6 × 10 sec of reperfusion/ischaemia. In the *in vivo* setting, several researchers have shown the ability of a 3 × 10-sec protocol to reduce infarct size after 30 min of regional ischaemia.^32^,^33^ Lim and co-workers^33^ compared a 3 × 10-sec and a 6 × 10-sec protocol and found that although both reduced infarct size, the 3 × 10-sec protocol was slightly more protective than the 6 × 10-sec protocol. Interestingly, Boengler and colleagues^34^ found that although both a 3 × 10-sec and a 5 × 5-sec protocol reduced infarct size, the 5 × 5-sec protocol seemed more robust in that it also exerted an infarct-sparing effect in aged and STAT3 knock-out mice (while the 3 × 10-sec protocol was inefficient in these models).

In the isolated heart perfusion set-up though, the 6 × 10-sec protocol is favoured, Kin *et al.*^35^ found that a 6 × 10-sec protocol was associated with an improved post-ischaemic systolic and diastolic function in the first minutes of reperfusion after 20 min of global ischaemia, in contrast to a 3 × 10-sec protocol, which proved ineffective. These findings are noteworthy, since they indicate an anti-stunning effect for postC (at least in mice).

Confirming these results, Morrison *et al.*^36^ applied a 6 × 10-sec protocol in their *ex vivo* preparation, which also elicited an increase in functional recovery, as well as a reduction in cardiac troponin I (TnI; a marker of cell damage) release. The murine model of postC, however, does not escape the experimental variability that is so common in postC research, as shown in Table 1.

## The rat heart

Despite considerable variability in protocols applied in both the mouse and rabbit hearts, postC was generally reported to be associated with a cardioprotective effect. Although the question as to the optimal postC intervention remains, it might not be that important, since the protection elicited seems to be robust in these animal species. The picture is, however, more complicated in the rat heart.

The first researchers to attempt postconditioning the rat heart were Kin and co-workers,^37^ who found in an *in vivo* model that a postC protocol of 3 or 6 × 10 sec applied immediately at the onset of reperfusion (after 30 min of regional ischaemia) led to a decrease in infarct size, creatine kinase (CK) activity, neutrophil accumulation in the AAR, as well as a decrease in oxidative-related damage [as measured by plasma malondialdehyde (MDA) levels] and superoxide anion generation. This first rat study also illustrated the importance of the immediate application of the intervention at the onset of reperfusion, since it was found that the postC intervention lost its protective effect when its implementation was delayed by one minute. Two other noteworthy observations were also made in this study: the infarct-sparing effect in the rat was less robust than had been described in the dog^7^ and rabbit;^14^ and the infarct-sparing effect of IPC in the rat heart was notably stronger than postC-associated protection.

Despite these observations, postC had been shown to be possible in the rat heart and since then, several studies have shown the efficacy of short (in the order of 10-sec) cycles of reperfusion and ischaemia.^25^,^26^,^38^ (Table 2). Confirming the observation that postC is not as robust as IPC,^37^ Tang and colleagues^26^ found that while postC could only protect after 30 min of coronary occlusion, IPC (12 × 2-min occlusion/reperfusion) could protect against infarct development after 45 and even 60 min of ischaemia.

**Table 2:** Postconditioning Of The Rat Heart By Applying 10-Sec Cycle Post-C Protocols

	*Experimental set-up*				*PostC protocol*	*Outcome*				
		*Ischaemia*					*Results*			
*Authors*	*Model*	*Type*	*Duration*	*Reperfusion duration*		*Parameter*	*Control*	*PostC*	*Difference (%)*	*Conclusion*
Penna *et al.*^25^	*Ex vivo* constant flow	GI	30 min	120 min	5 × 10 sec	IFS	65 ± 4%	22 ± 4%	↓ 66	Protective
						LDH release	1950 ± 100	656 ± 93	↓ 66	
					15, 20, 25, 30 sec reperfusion with 20, 15, 10, 5 sec ischaemia	IFS		20 ± 2%	↓ 69	Protective
						LDH release		650 ± 60	↓ 66	
Penna *et al.*^25^	*Ex vivo* constant pressure	GI	30 min	120 min	5 × 10 sec	IFS	59 ± 5%	46 ± 2%	↓ 22	Protective
						LDH release	1842 ± 77	686 ± 34	↓ 63	
					15, 20, 25, 30 sec reperfusion with 20, 15, 10, 5 sec	IFS		45 ± 2%	↓ 24	Protective
						LDH release		675 ± 59	↓ 63	
Tang *et al.*^26^	Conscious rat	RI	30 min	24 h	6 × 30 sec	IFS	54.4 ± 2.3%	55.8 ± 3.5%	NS	Non-protective
					6 × 10 sec			36.1 ± 5.0%	↓ 34	Protective
					20 × 10 sec			28.9 ± 4.9%	↓ 47	Protective
					60 × 10 sec			57.3 ± 5.4%	NS	Non-protective
			45 min		20 × 10 sec		62.2 ± 2.4%	55.4 ± 2.4%	NS	Non-protective
			60 min		20 × 10 sec		72.7 ± 2.2%	71.4 ± 3.4%	NS	Non-protective

RI: regional ischaemia; IFS: infarct size; NS: non-significant; GI: global ischaemia; LDH: lactate dehydrogenase (U/g wet weight).

Application of longer reperfusion/ischaemia cycles (30 sec) also seem to be effective in eliciting protection, contrary to the observations reported by Tang *et al.*^31^ (Table 2). Manintveld and colleagues^39^ found that 3 × 30-sec cycles of reperfusion/ischaemia applied after 45 or 60 min of coronary occlusion in an *in vivo* model reduced infarct size. In their study, postC (3 × 30-, 3 × 5- and 3 × 15-sec cycles of reperfusion/ischaemia) could not confer cardioprotection after 90 or 120 min of ischaemia, and surprisingly, significantly aggravated infarct size when applied after 30 or 15 min of ischaemia. These latter observations are contrary to expectation, but the authors argued that it illustrates that the duration of sustained ischaemia could also determine the efficacy of a postC intervention.

Intriguingly, Tillack *et al.*^40^ successfully employed a 3 × 30-sec protocol to decrease infarct size after 30 min of regional ischaemia in their *in vivo* model. This difference in outcome between these two similar experimental set-ups still remains to be explained. Bopassa and co-workers^41^ also found a 3 × 30-sec protocol to be cardioprotective in their isolated heart set-up, since it was associated with an increase in functional recovery, as well a reduction in the levels of markers of myocardial necrosis [lactate dehydrogenase (LDH), CK and TnI] in the coronary effluent. An intriguing difference in their protocol was the administration of one-minute reperfusion before the application of postC (in constrast to most other protocols in which postC was immediately applied).

Another recent study also demonstrated the cardioprotective ability of the 3 × 30-sec protocol, but in this case in the isolated, working rat heart.^42^ These workers found that this intervention preserved collagen content, decreased free radical production, converted reperfusion arrhythmias into normal rhythm and increased functional recovery after four hours of hypothermic (4°C) cardioplegic arrest – illustrating the potential of postC in the setting of open-heart surgery.

Interestingly, and surprisingly, some studies have been reported which applied a postC intervention consisting of a single cycle of ischaemia, more than a minute in duration, after several minutes of reperfusion (Table 3). These mould-breaking studies have only been done in the setting of reperfusion arrhythmias and fibrillation.

**Table 3:** Unique Post-C Protocols Applied In The Rat Heart To Elicit Protection Against Reperfusion Arrhythmia And Fibrillation, As Well As Post-C Protocols Reported To Be Cardioprotective In The Pig Heart. Each Of These Porcine Studies Utilised Different Post-C Protocols And Experimental Designs

	*Experimental set-up*					*Outcome*				
		*Ischaemia*					*Results*			
*Authors*	*Model*	*Type*	*Duration*	*Reperfusion duration*	*PostC protocol*	*Parameter*	*Control*	*PostC*	*Difference (%)*	*Conclusion*
***Postconditioning the rat heart: unique protocols***										
Galagudza *et al.*^27^	*Ex vivo*	RI	30 min	30 min	15 min reperfusion + 2 min GI	Ventricular fibrillation	During postC ischaemia: conversion of VF; onset of stable, regular rhythm after postC			Anti-fibrillation effect
Sasaki *et al.*^28^	*Ex vivo* (working model)	GI	15 min	20 min	1 min reperfusion + 5 min GI	Arrhythmias	Termination of ventricular arrhythmia, thus shorter duration of arrhythmia in postC hearts			Antiarythmic effect
***Recently reported protective postC protocols in the pig heart***										
Jiang *et al.*^29^	*In vivo*	RI	75 min	180 min	3 × 30 sec	IFS	45 ± 5%	12 ± 4%	↓ 73	Protective
Skyschally *et al.*^30^	*In vivo*	Low flow	90 min	120 min	6 × 20 sec	IFS	33.8 ± 4.4%	19.5 ± 2.9%	↓ 42.3	Protective
Zhao *et al.*^31^	*In vivo*	RI	180 min	120 min	6 × 10 sec	IFS	98.5%	76.1%	↓ 22.7	Protective
						No-reflow area	81.3%	54.3%	↓ 33.2	
						HR	108 ± 6	107 ± 9	NS	
						LVSP	109 ± 3	111 ± 2	↑ 2	
						LVEDP	6.1 ± 1.6	4.9 ± 1.9	↓ 19.7	
						+ dp/dt	2287 ± 551	2759 ± 492	↑ 21	
						− dp/dt	2112 ± 242	2319 ± 183	↑ 10	
						CO	1.34 ± 0.25	1.94 ± 0.31	↑ 44.78	

RI: regional ischaemia; IFS: infarct size; NS: non-significant; GI: global ischaemia; VF: ventricular fibrillation; LVSP: left ventricular systolic pressure (mmHg); LVEDP: left ventricular end-diastolic pressure (mmHg); ± dp/dt: maximal change rate of left ventricular pressure rise and fall (mmHg/sec); CO: cardiac output (l/min).

Two recent studies have further highlighted the irregularity and variability of the outcome of postconditioning, specifically in the rat heart. Dow and Kloner^43^ attempted to postcondition the *in vivo* rat heart after either 30 or 45 min of regional ischaemia. They applied various protocols: 4 × 10-, 4 × 20-, 8 × 30- and 20 × 10-sec cycles of reperfusion/ischaemia. None of these protocols could reduce infarct size, despite the successful application of IPC, and the previous findings in their laboratory that postC does reduce ventricular arrhythmias.^44^ One possible explanation for these findings may lie in the fact that they used female rats.

Crisostoma *et al.*^45^ found that although the female rat heart can be postconditioned (postC protocol: 6 × 10-sec cycles), this protection was dependent on the degree of ischaemic injury. In their *ex vivo* model, male hearts were postconditioned after 20 and 25 min of global ischaemia, while female hearts could only be protected after 20 and not 25 min of ischaemia.

Kaljusto and co-workers^46^ also experienced problems postconditioning the rat heart. In their study they investigated rats and mice, both *in vivo* and *ex vivo*, with the goal of developing a robust postC protocol. Although they could demonstrate cardioprotection in mice, only in one laboratory (of two) were they able to elicit cardioprotection in the *in vivo* rat model (with a protocol of 3 × 10-sec reperfusion/ischaemia after 30 min of regional ischaemia). In the isolated rat heart they investigated various protocols: 3 × 10-, 3 × 30- or 2 × 60-sec cycles of reperfusion/ischaemia following 30 min of global ischaemia; while after 40 min regional ischaemia they applied a 3 × 10-sec, as well as a 6 × 10-sec cycle protocol. They could, however, not induce an infarct-sparing effect with any of these protocols.

The rat heart can indeed therefore be postconditioned, although the precise optimal protocol with which cardioprotection can be achieved is still an unresolved question. To date, we do not have an explanation for the reported variability in outcomes. It could be that with regards to the rat heart, there are confounding factors that have not yet been identified. In our laboratory, for example, we found that postC only elicited an infarct-sparing effect when it was applied within a narrow temperature range around 37°C.^47^

## The pig heart

Despite the inconsistent results found in the rat heart, postC experiments in the pig heart seemed to cause the most concern. The first article published on postC in the pig heart did not report success: Schwartz and Lagranha^48^ applied a protocol of 3 × 30-sec reperfusion/ischaemia in their *in vivo* porcine heart model of regional ischaemia (30 min of coronary occlusion). This protocol could, however, not limit infarct size, although IPC could confer cardioprotection in this model. These initial findings raised questions as to whether all mammal species could be postconditioned.

Iliodromitis *et al.*^49^ subsequently evaluated the efficacy of the protocol applied by Schwartz and Lagranha.^48^ They compared a 4 × 30-sec protocol with an 8 × 30-sec cycle protocol, applied after 60 min of coronary ligation, in the *in vivo* model. The 8 × 30-sec protocol elicited an infarct-sparing effect. The authors speculated that the total time of postC intervention (four vs eight minutes) might explain these differences, since in the longer protocol, the heart was exposed to the protective postC trigger(s) for a more substantial period of time. Doubt is, however, cast on the importance of the total time of postC intervention by studies that have recently been reported using short periods of postC (Table 3).

Unfortunately, the reason(s) why the initial study in the porcine model could not show cardioprotection is still unknown. Should the findings made by Manintveld *et al.*^39^ namely, that a too-short ischaemic duration could render postC non-cardioprotective be extrapolated to pigs, it might also be that Schwartz and Lagranha^48^ applied a suboptimal period of regional ischaemia (too short) to successfully elicit postC protection. It is noteworthy that all the studies that have reported cardioprotective postC in pigs applied a longer index ischaemia (see Table 3).

## Considerations to remember when postconditioning

The many studies done thus far on postconditioning clearly show that it is a very real cardioprotective intervention that can be induced in all species tested: dog, rabbit, mouse, rat and pig. The numerous variations in experimental set-up, postC protocol applied and postC efficacy, even within species, are however notable and demonstrate the variability of the phenomenon. Therefore, the existence of a reperfusion intervention that can salvage myocardium is undisputable, but reliable stimulation of this effect by the application of cycles of reperfusion and ischaemia (postC) seems to be hampered by various confounders (many of which might still be undefined).

Several variables can, however, be identified, which seem to be of importance in determining the efficacy of a postC protocol in animal models:

● The animal species being tested. Despite the phenomenon being described in all species, it does seem as if it is more difficult to elicit postconditioning in the rat.^43^,^46^ In a recent review, Vinten-Johansen *et al.*^50^ speculated that the observed species differences may be due to differences in the rate and degree of ischaemia/reperfusion injury development in different animal species. These parameters are determined by factors such as myocardial metabolism, endogenous antioxidant defences and the role of inflammatory cells during reperfusion.^50^
● Gender could also play a role. Since only one study has been reported on this subject,^45^ there is a need for more research into the importance of this variable.
● Differences in the endpoints utilised also complicate the interpretation of results. Supplementation of the routinely used endpoints of functional recovery and infarct size with standard biochemical parameters indicative of damage (such as creatine kinase and lactate dehydrogenase release) could facilitate comparison of results.
● The time lapse between the end of sustained ischaemia and the actual onset of the postC intervention. Although some researchers have successfully applied a postC intervention after a period of reperfusion,^14^,^41^ postC is generally applied as soon as possible after ischaemia.
● Duration of the reperfusion/ischaemia cycles. Although these cycles are rarely more than 60 seconds, it does seem as if the postC intervention is quite sensitive to even small changes in cycle duration.
● The number of cycles. The total duration of postC intervention (determined by both the duration and numbert of cycles) might also be of importance,^49^ although the literature does show considerable variability in this regard.
● The duration of sustained ischaemia. This variable might not be as straightforward as expected^39^ and needs further investigation. If the efficacy of postC is partly determined by the duration of prior ischaemia, it could complicate the implementation of postC in the clinical setting.

## Postconditioning in cell culture

Although animal models are very useful for investigation of a phenomenon in a natural physiological setting, the utilisation of cell cultures is essential for determination of the mechanisms involved. This approach has also been recruited for the investigation of postC (Table 4). Although only three studies have been published on postC in isolated cells and cell culture, these studies all report that postC elicits protection against hypoxia/reoxygenation damage. Interestingly, all three studies utilised similar postC protocols (two or three cycles of five minutes’ hypoxia/reoxygenation) to elicit this protection. The increase in cycle duration (from seconds in animals to minutes in cells) could be explained by the difference in metabolic rate: the metabolic rate of isolated cells and cell cultures is substantially lower than that of hearts *in vitro* or *in vivo*.^51^

**Table 4:** Summary Of Studies Reported On The Feasibililty Of Post-C In Cellular Preparations

*Authors*	*Cell type*	*Hypoxia*	*Reoxygenation*	*PostC*	*Comments*
Sun *et al.*^51^	Neonatal rat cardiomyocytes	3 h (hypoxic incubator: 95% N_2_ 5% CO_2_)	6 h	3 × 5 min (switching between normoxic and hypoxic incubators)	PostC reduced cell death (PI staining and LDH release)
					Reduced ROS generation
					Reduced intracellular and mitochondrial Ca^2+^
Sun *et al.*^52^	Neonatal rat cardiomyocytes	3 h	6 h		PostC reduced apoptosis and DNA fragmentation
					Associated with: ↓ superoxide generation, ↓ JNK and p38 MAPK activity, ↓ TNF-α release, ↓ caspase 3 and 8 activity, ↓ Bax
Zhao *et al.*^53^	H9c2 cardiac muscle cells	8 h	3 h	3 × 5 min	PostC reduced number of apoptotic cells and DNA fragmentation
					Associated with: ↓ cytochrome C release, ↓ loss in mitochondral membrane potential and inhibition of mPTP, ↓ Bax and ↑ Bcl-2 in mitochondria, ↑ phospho-PKB/Akt and phospho-ERK in isolated mitochondria
Wang *et al.*^54^	Isolated rat cardiomyocytes	2 h	3 h	2 × 5 min (switching between normoxic and hypoxic incubators)	PostC increased cell viability (assessed with trypan blue staining) and decreased LDH release and apoptosis
					Associated with reduced ONOO– generation following hypoxia/reoxygenation

PI: propidium iodide; LDH: lactate dehydrogenade; ROS: reactive oxygen species; JNK: c-Jun NH_2_-terminal kinase; p38 MAPK: p38 mitogenactivated protein kinase; TNF-α: tumour necrosis factor-α; mPTP: mitochondral permeability transition pore; PKB: protein kinase B; ERK: extracellular signal-regulated kinase; ONOO–: peroxynitrite.

## Mechanisms behind postconditioning

Despite the variability in the postC protocols utilised, several gains have been made in understanding the mechanism of postC-associated protection – especially since the large body of work done on IPC has established a firm base for investigations into cardioprotective interventions. Although the mechanisms whereby postC and IPC elicit cardioprotection is beyond the scope of this review, the role-players identified in IPC that have influenced postC research will briefly be discussed, as well as current views regarding the mechanism of postC.

## Ischaemic preconditioning: setting the stage for postconditioning

For a detailed review on the mechanisms implicated in IPC, see Yellon and Downey.^6^ The intracellular mechanisms at work in IPC have been conceptualised as a ‘trigger–mediator–end-effector’ pathway. The triggering phase entails the activation of protective cascade(s) during the actual preconditioning intervention, prior to ischaemia. Some triggers identified in IPC have also been implicated in postC, for example, adenosine,^55^ bradykinin^56^ and free radicals.^57^,^58^ The borders dividing ‘triggers’, ‘mediators’ and ‘end-effectors’ have however become blurred, since some molecules and pathways have been implicated as triggers, and also as mediators and end-effectors.

In this regard, both the MEK 1/2 (MAPK/ERK kinases)–ERK 1/2 (extracellular signal-regulated kinase)^59^ and the PI3-kinase (phospatidylinositol 3-kinase)–PKB (protein kinase B)/Akt pathways^60^,^61^ have been implicated in the triggering phase of IPC, as well as during reperfusion after sustained ischaemia.^62^ In fact, Hausenloy *et al.*^63^ reported that activation of both pathways during reperfusion is necessary for IPC to confer protection. The possible downstream end-effector of these pathways, the mitochondrial permeability transition pore (mPTP),^64^ has also been implicated before and after ischaemia. Brief, low-conductance opening of the mPTP during the conditioning stage of IPC (ie, prior to ischaemia) has been implicated in protection, possibly by mediating ROS-dependent protection^65^ (although this has been challenged by Halestrap *et al.*^66^). It has also been shown that IPC inhibits mPTP opening during reperfusion,^67^,^68^ thereby eliciting cardioprotection.

**Fig. 1.**
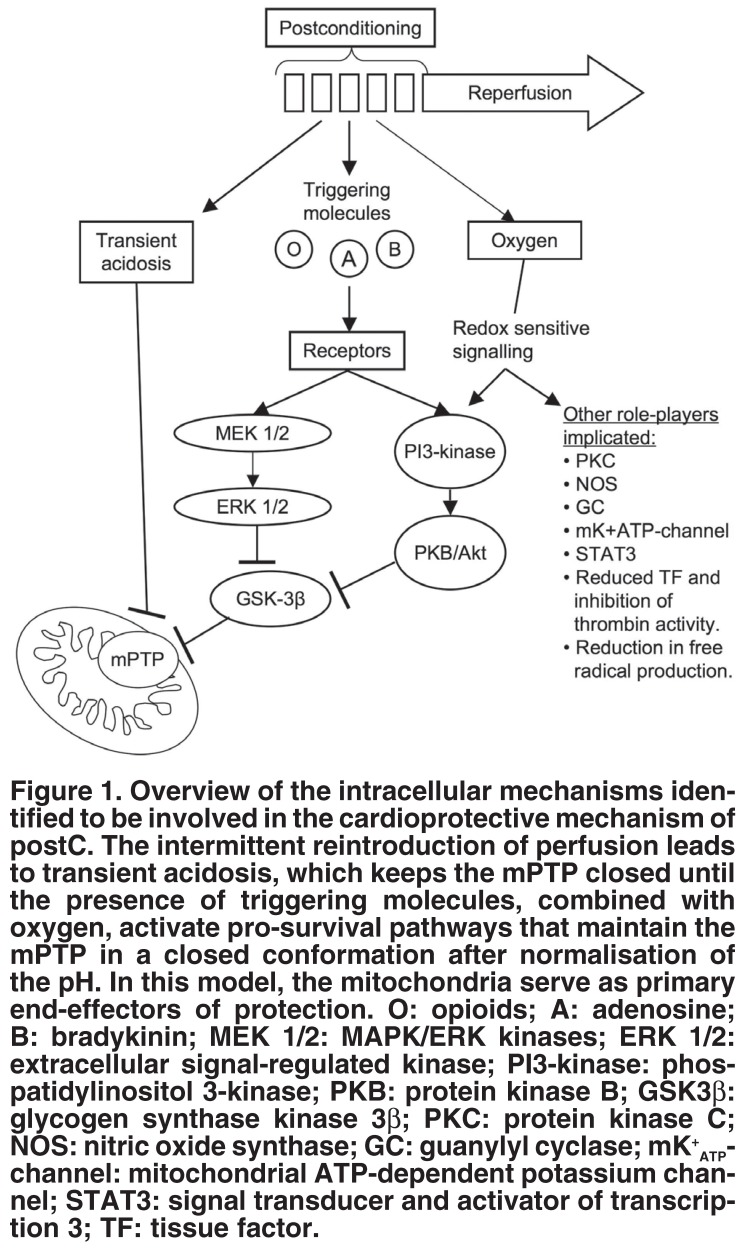
Overview of the intracellular mechanisms identified to be involved in the cardioprotective mechanism of postC. The intermittent reintroduction of perfusion leads to transient acidosis, which keeps the mPTP closed until the presence of triggering molecules, combined with oxygen, activate pro-survival pathways that maintain the mPTP in a closed conformation after normalisation of the pH. In this model, the mitochondria serve as primary end-effectors of protection. O: opioids; A: adenosine; B: bradykinin; MEK 1/2: MAPK/ERK kinases; ERK 1/2: extracellular signal-regulated kinase; PI3-kinase: phospatidylinositol 3-kinase; PKB: protein kinase B; GSK3β: glycogen synthase kinase 3β; PKC: protein kinase C; NOS: nitric oxide synthase; GC: guanylyl cyclase; mK^+^_ATP_- channel: mitochondrial ATP-dependent potassium channel; STAT3: signal transducer and activator of transcription 3; TF: tissue factor.

It is especially these role players that have been implicated in IPC during reperfusion, ie, the MEK 1/2–ERK 1/2 and PI3-kinase–PKB/Akt pathways, as well as the mPTP, which seem to be vitally important in postC-mediated protection. The mechanism is summarised in Fig. 1 and is discussed in the following section (also reviewed by Zhao and Vinten-Johansen,^69^ Hausenloy and Yellon,^70^ and Tissier *et al.*^4^).

## Postconditioning

The intermittent initial reperfusion associated with postC leads to a state of transient acidosis,^13^ inhibiting the formation of the mPTP,^71^ which has been implicated in cell death. Concurrent with the maintenance of acidosis, intermittent reperfusion also causes the retention of triggering molecules (such as bradykinin, 72 opioids^73^ and adenosine^17^,^35^) within the myocardium, which then activates their respective receptors to activate a protective signalling pathway(s). This pathway(s) seems to be redox sensitive, since the administration of free radical scavengers either before or during the postC intervention abrogates its cardioprotective effects, implying a vital role for oxygen delivery during the postC intervention.^72^,^74^,^75^ Although it is redox sensitive, postC is also associated with a reduction in free radical generation, compared to control hearts.^7^,^11^,^37^

As has been speculated by others,^72^,^74^,^75^ the precise effect of free radicals in reperfusion might be dependent on the ROS species, amount, timing and cellular compartment involved. In this regard, it is noteworthy that opening of the mitochondrial ATP-dependent potassium channel (mK^+^_ATP_ channel), which has been shown to be associated with postC protection,^14^ has been implicated in the generation of triggering free radicals,^72^,^74^ as well as the activation of postC-associated protective pathways.^76^

Following these triggering events, postconditioning recruits the so-called RISK (reperfusion injury salvage kinase) pathway, which includes both the MEK 1/2–ERK 1/2^13^,^14^,^18^,^36^ and PI3-kinase–PKB/Akt^13^,^14^,^36^,^40^,^77^ pathways (for a review on RISK in ischaemia/reperfusion see reference 78). These kinases in turn inhibit the opening of the mPTP via the inhibitory phosphorylation of GSK3β (glycogen synthase kinase 3β).^21^,^40^,^41^,^77^

Other signalling kinases have also been implicated in the transduction of pro-survival signals, such as protein kinase C (PKC),^74^ nitric oxide synthase (NOS) (which has been shown to be downstream of PI3-kinase activation),^14^,^25^,^39^,^77^,^79^ guanylyl cyclase (GC)^17^,^25^ and protein kinase G.^80^ The end-result is that by the time the pH has normalised in the cells, the survival kinases have been activated to ensure that the mPTP remains closed.^81^ Keeping the mPTP in a closed conformation is vital, since opening of the pore favours cell death – via either apoptosis (mediated by released cytochrome C and outer membrane rupture), or necrosis [due to a loss of mitochondrial membrane potential leading to the uncoupling of oxidative phosphorylation and an eventual loss of adenosine triphosphate (ATP)].^82^ Signal transducer and activator of transcription 3 (STAT3)^34^ has also been implicated in postC protection.

Besides these intracellular mechanisms, the initial studies on postC in the *in vivo* set-up also reported that postC attenuated the inflammatory response, as observed in the reduction in tissue oedema and neutrophil accumulation in the area at risk.^7^,^10^,^11^,^37^ These latter two observations could be contributory to the reduction in no-reflow area associated with postC.^31^ PostC has also been shown to be associated with a reduction in the expression of tissue factor (TF) and the inhibition of thrombin activity in the area at risk.^29^ PostC cardioprotection has also been linked to the preservation of coronary artery endothelial function.^7^

Postconditioning, therefore, clearly recruits various mechanisms to exert its cardioprotective effects. It is this ‘pleiotropic’ effect of the postC intervention that renders it effective in exerting cardioprotection, and also increases its appeal as a possible intervention in the clinical setting of myocardial ischaemia/reperfusion.

## To the bedside: postconditioning in the clinical setting

In light of the above-discussed variations in the efficacy of postC in the laboratory, one would be forgiven for expecting the phenomenon to remain in the realm of laboratory science for the time being.

The potential of postC to protect human tissue has, however, been demonstrated in two laboratory-based studies. By monitoring flow-mediated dilation of the brachial artery as functional endpoint, Loukogeorgakis *et al.*^83^ demonstrated that both 3 × 30-sec and 3 × 10-sec cycles of ischaemia/reperfusion can be used to decrease transient functional damage after a 20-min ischaemic insult on the forearm of test subjects. Sivaraman and co-workers^84^ investigated the ability of a 4 × 30-sec and 4 × 60-sec protocol to protect isolated human atrial trabeculae from functional damage following 90 min of simulated ischaemia (paced at 3 Hz), and 120 min of simulated reperfusion (paced 1 Hz). They found that only 4 × 60 sec induced protection, which was dependent on PI3-kinase and MEK 1/2 activity (in agreement with animal model studies).

Even prior to these studies, the existence of a cardioprotective intervention applicable at the clinically relevant time-point of reperfusion has energised research into the possibility of translating this phenomenon into a clinically viable therapy.

## PostC: a mechanical intervention

In 2005, Laskey^85^ published a pilot study in which he investigated the effects of a preconditioning-like intervention applied in reperfusion. This study focused on patients presenting with an acute myocardial infarct, receiving percutaneous coronary intervention. In all patients, flow greater than TIMI grade 0–1 was established by minimum intervention in the infarct-related artery. Following initial reperfusion, patients received either a conditioning intervention (ie, two 90-sec balloon inflations inthe stenotic artery, divided by three to five min of reperfusion), or usual care, which entailed a single 90-sec inflation at the same time as the second inflation in the conditioned group. All patients experienced relief of angina, a decrease in stenosis to less than 10% and coronary flow greater than TIMI grade 2.

In this study, Laskey^85^ found that the preconditioning-like stimulus was associated with favourable changes in electrocardiographic and coronary haemodynamic markers. Although it is questionable if this study really applied a true postC intervention, it certainly illustrated the potential for postconditioning in humans. This potential for postC protection was confirmed by a retrospective analysis of patients who had received angioplasty after presenting with myocardial infarction.^86^ It was found that four or more balloon inflations at reperfusion were associated with less peak creatine kinase release than when between one and three inflations were applied.

Four studies have been reported that investigated postC in humans in the clinical setting. Staat and co-workers^87^ applied a postC protocol of four cycles of one-minute reperfusion/ischaemia at the onset of reflow, after angioplasty. This was achieved by inflating and deflating the angioplasty balloon upstream of the implanted stent (to avoid damaging the stent, as well as to prevent thrombus embolisation). This intervention decreased infarct size (as measured by the area under the creatine kinase curve) after 72 hours of reperfusion, illustrating the feasibility and cardioprotective ability of postC in the human heart.

The question whether postC permanently protects tissue or merely delays damage was adressed by Yang *et al.*^88^ They applied a postC protocol of 3 × 30 sec of reperfusion/ischaemia in patients undergoing PCI, by deflating and inflating the angioplasty balloon. They confirmed the reduction in infarct size observed by Staat *et al.*^87^ but by using nuclear imaging they also observed a sustained decrease in infarct size after seven days of reperfusion.

Applying a similar protocol, Ma and co-workers^89^ found that postC was associated with a decrease in blood levels of MDA and CK – illustrating a decrease in free radical-mediated cell injury. They also reported an increase in microcirculation reperfusion, peripheral artery endothelial function and left ventricular wall motion (measured eight weeks after PCI). Recently Luo *et al.*^90^ demonstrated that a 3 × 30-sec postC protocol (administered by opening and closing the aortic clamp) in the setting of cardiac surgery (specifically, valve replacement) was associated with a reduction in myocardial necrosis (determined by measuring CK–MB levels).

The few studies that have been done on postC in the clinical setting therefore indicate that the human heart can be postconditioned.

## Pharmacological postconditioning

The sensitivity of the postC intervention for various factors (as illustrated in the laboratory), as well as the risks that are associated with the manipulation of coronary flow in highrisk patients with probable unstable atherosclerotic lesions are factors that could limit the application of ischaemic postC in the clinical setting. A primary focus in postC research is therefore to identify pharmacological mimetics, which could be administered in reperfusion to stimulate a more reliable and risk-free form of cardioprotection. In this respect, various candidate compounds have come to light, such as adenosine^35^ and its receptor agonists,^91^ bradykinin,^71^,^91^ B-type natriuretic peptide,^92^ volatile anesthetics such as isoflurane^20^ and others.^4^

A recent study has shown the potential of pharmacological postC to confer cardioprotection in the human heart. Jin and co-workers^93^ reported that administration of adenosine (1.5 mg/kg) within one minute of aorta cross-clamp removal after heart valve replacement surgery was associated with a significant reduction in cardiac troponin I release at 12 and 24 hours after cross-clamp removal.

## Conclusion

The description of the protective ability of postC, an intervention applied during reperfusion, has indeed energised research on the effects of ischaemia/reperfusion and myocardial salvage during reperfusion after the alleviation of ischaemia. In this article, a systematic and critical overview was given of the application and success of postC in several animal models. A close look at this promising intervention reveals various practical considerations that should be taken into account when designing a study on postC, and which are therefore important if postC is to progress convincingly from basic science to standard clinical care.

In fact, the variability in postC protocols applied is disturbing and either indicates a robustness in the protection of postC (since various protocols elicit protection in the same species), or a lack of reproducibility between studies (since different laboratories found it necessary to utilise different protocols to elicit protection). This lack of consistency in experimental setup and efficacy is a problem that could hamper future research, especially into the clinical applicability of postC. Multi-centre laboratory studies (such as reported by Kaljusto *et al.*^46^) could be a way to address this problem.

Despite the experimental problems experienced, a great deal of insight has been obtained into the mechanisms of postC cardioprotection. PostC manoeuvres have also been shown to confer a degree of protection in humans, illustrating clinical promise. Taken together, these observations indicate that a reperfusion-based intervention, decreasing ischaemic/reperfusion damage through alterations in the intracellular milieu is feasible and does exist.

In the light of the variability in ischaemic postC, as demonstrated in various laboratory studies, we suggest that pharmacological reperfusion therapies, harnessing the mechanisms associated with ischaemic postC, is the best way forward in translating laboratory myocardial salvaging to clinical ischaemia/reperfusion treatment.
